# Psychometric properties of the quality nursing care scale-turkish version: a methodological study

**DOI:** 10.1186/s12912-022-01153-0

**Published:** 2022-12-27

**Authors:** Anita Karaca, Leyla Kaya, Gizem Kaya, Arzu Kader Harmanci Seren

**Affiliations:** 1 Department of Nursing, Faculty of Health Sciences, Biruni University, Istanbul, Turkey; 2 Zeynep Kamil Women and Children Diseases Training and Research Hospital, Istanbul, Turkey; 3 Department of Nursing, Faculty of Health Sciences, Fenerbahce University, Istanbul, Turkey

**Keywords:** Nursing care, Quality of care, Perception, Psychometrics

## Abstract

**Aim:**

To analyze the psychometric properties of the Quality Nursing Care Scale in Turkish Language.

**Background:**

The quality of health services and nursing care effectively improves safe patient outcomes and reduces costs in healthcare organizations. There is a need for valid and reliable tools in order to use for evaluating the quality of nursing care.

**Methods:**

The methodological and cross-sectional study included 225 nurses working in a research and training hospital. Content validity, construct validity, item analysis, and internal consistency analysis were used.

**Results:**

The content validity index of the scale was 0.96. The item-total score correlation values of the items were 0.72 and higher. The factor loads of the items ranged from 0.42 to 0.90. Different from the original scale, Turkish form consisted of three sub-dimensions. The fit indices were acceptable or very good. The Cronbach’s alpha internal consistency coefficient was 0.99.

**Conclusion:**

The Quality Nursing Care Scale was valid and reliable with its three-factor structure in Turkish Language. It may be used for measuring the quality of care in the aspects of nurses.

## Background

Today, hospitals have focused on improving care services to meet consumers’ expectations [1, 2]. The World Health Organization (WHO) reports that approximately 15% of the total deaths (5.7–8.4 million deaths per year) in low- and middle-income countries are due to poor quality care. According to the WHO, quality of care is related to whether the health services provided to individuals and society achieve the desired health outcomes. Thus, health services must be delivered safely, effectively, timely, and efficiently and be equitable and people-centered [3].

Care is at the center of nursing knowledge, skills, and practice. The effectiveness of the care provided plays a role in patient treatment compliance. Improving a healthy individual's health or restoring a sick individual's health is possible through effective nursing care. Therefore, individuals' quality of life can be enhanced through care practices. The hospitalization duration for patients receiving qualified nursing care is also shortened [4]. Nurses are the most critical human resource in providing quality healthcare [5–7]. Quality nursing care is one of the factors that can increase patient satisfaction and positively affect patients' recovery processes. In addition, quality nursing care is highly effective in achieving targeted patient outcomes, protecting patients from possible dangers, preventing undesirable consequences, and ensuring patient safety [2, 8, 9].

Patients and their relatives, hospital administrators, and nurses who provide services may assess the quality of nursing care in their aspects. When evaluating nursing care, patients and relatives mainly focus on their communication with the nurses and whether they can get answers to the questions they ask the nurses. Hospital or nursing managers generally focus on efficiency and cost-effectiveness when evaluating the quality of care. This shows that participants who take on different roles have different knowledge, opinions, and values. Each stakeholder assesses the quality of care in line with their knowledge, belief, and value. Therefore, nurses’ evaluations of the care they provide are critical in improving and developing the quality of nursing services [1, 10].

Nurses are frontline healthcare professionals who identify, plan, and evaluate patients’ needs, advocate for patients, administer medications and treatments and ensure their comfort [3, 11]. It is also essential for psychiatric and pediatric clinics, where patients may not evaluate the quality of their care sufficiently in their aspects. Also, it is challenging for patients and other service recipients to evaluate the technical competencies, knowledge levels, and skills of care professionals [12, 13].

In the literature, limited studies have evaluated the adequacy of nurses’ nursing care [11, 14, 15]. In addition, many published articles have assessed the quality of nursing care from the patient’s perspective [16–18]. However, Lynn et al. [14] stated that evaluating the quality of patient care would be incomplete without considering nurses’ perspectives. To provide quality care, nurses must be sure of the care provided before all other actors. It allows for developing action plans to measure the quality of care perceived by nurses, strengthen trust in care, and identify potential areas for improvement. It can also help nurses better understand patients' real needs and develop strategies for care [19]. Thus, this study aimed to investigate the psychometrics of the Quality Nursing Care Scale among nurses in the Turkish Language.

## Methods

### Aim

This study aimed to evaluate the psychometrics of the Quality Nursing Care Scale in Turkish.

### Study design, setting and sample

This was a methodological and cross-sectional study conducted at a training and research hospital. All nurses working in the hospital's inpatient wards were included in the study. The study sample consisted of 225 nurses accepted to participate in the study. The mean age of the participant nurses was 33.81 (SD = 7.70) years. Nurses’ professional and unit experiences were 8.95 (6.94) and 4.29 (4.37) years, respectively. Among the nurses, 86.7% were women, 61.8% were married, and 59.1% were undergraduates: they primarily were working at surgical units (40.0%), both day and night shifts (78.2%). Most of the nurses were working 46 or more hours weekly (73.8%) and caring for 11 or more patients in each change. Also, 66.2% of them were working evening and night shifts six or more times in a month (Table 1).

**Table 1:** Personal and professional characteristics (*N* = 225)

Variables	Categories		
		***n***	**%**
Mean age (standard deviation)	33.81 (7.71) years		
Mean professional experience (standard deviation)	8.95 (6.94) years		
Mean unit experience (standard deviation)	4.29 (4.37) years		
Sex	Female	195	(86.7)
	Male	30	(13.3)
Marital status	Single	86	(38.2)
	Married	139	(61.8)
Education	High school	34	(15.19)
	Associate degree	24	(10.7)
	Bachelor’s degree	133	(59.1)
	Graduate	34	(15.1)
Unit	Surgical	90	(40.0)
	Medical	79	(35.1)
	Intensive Care Unit	56	(24.9)
Working type	Night shifts	10	(4.4)
	Day shifts	39	(17.3)
	Day and night shifts	176	(78.2)
Weekly working hours	45	59	(26.2)
	≥ 46	166	(73.8)
Average number of cared patients in a shift	1–5	80	(35.6)
	6–10	48	(21.3)
	≥ 11	97	(43.1)
Number of evening and night shifts in a month	None	32	(14.2)
	1–5	44	(19.6)
	≥ 6	149	(66.2)
Income	Low	149	(66.2)
	Equal or high	76	(33.8)
Satisfying with hospital	No	97	(43.1)
	Not sure	54	(24.0)
	Yes	74	(32.9)
Satisfying with unit	No	88	(39.1)
	Not sure	50	(22.2)
	Yes	87	(38.7)
Satisfying with working conditions	No	145	(64.4)
	Not sure	40	(17.8)
	Yes	40	(17.8)
Satisfying with salary	No	187	(83.1)
	Not sure	28	(12.4)
	Yes	10	(4.4)

### Procedure

The study followed the required scale adaptation steps according to the International Test Commission and Consensus-based Standards for the Selection of Health Measurement Instruments (COSMIN) guidelines [20–22]. Translating the items and measuring the content validity was the first step. Secondly, item-total score correlations of the items were calculated. Then construct validity was tested. Lastly, the internal consistency of the scale was analyzed.

### Data collection instruments

Research data were collected online (Google Forms) between September and November 2021. The online surveys were sent online to the nurses working in the institution. They were asked to fill out the forms. Only those who filled out the informed consent form could access the survey, and only the researchers could access the results.

The literature recommends reaching out to individuals 5–10 times the number of items in reliability and validity studies [23]. Since the number of items in the scale to be validated and tested for reliability was 38, researchers aimed at reaching a sample of at least 190 people. The study sample consisted of 225 nurses working at the time of the study. The study data were collected using a questionnaire containing questions to determine personal and professional characteristics and the Turkish version of the Quality Nursing Care Scale.

### Information form

It was a form consisting of 18 questions that sought data on the age, gender, marital status, and education level of the nurses participating in the research, the unit they worked in, their working style, their working hours as a nurse, their monthly duty hours, their perception of income, their satisfaction with the institution, unit, working conditions, and salary they received.

### Turkish version of quality nursing care scale

The Quality Nursing Care Scale (QNC) is a 5-point Likert-type tool developed by Liu et al. [10]. The scale consisted of six sub-dimensions and 38 items in its original form. The sub-dimensions were entitled Physical environment (six things), Staff characteristic (eight items), Precondition (seven items), Task-orientated activities (six items), Human-orientated activities (five items), and Patient outcomes (six things). The internal consistency coefficient of the scale was 0.96. The answer categories were graded between "strongly agree" (5) and "strongly disagree" (1). High scores indicate higher quality nursing care, and low scores indicate lower quality nursing care.

### Ethical considerations

The researchers got permission from the original work's owner to adapt the tool into Turkish. The original scale's author also confirmed that the scale had not been previously adapted to Turkish. Approval of a university hospital's Clinical Research Ethics Committee, dated 05.05.2021 and numbered 98, was obtained. Before data collection, formal written permission was obtained from the hospital administration. Only nurses who agreed to participate in the study and filled out the online informed consent form were included.

### Statistical analysis

Data were analyzed via Jamovi, an R-based open statistical software [24]. First, the Davis technique was used to analyze the content and content validity in the study. Second, Pearson correlation analysis was used for item analysis. Third, Kaiser–Meyer–Olkin and Bartlett's tests were used to evaluate sample adequacy. Fourth, exploratory factor analysis was used to determine the construct of the Turkish version. Extraction model was principal axis factoring and rotation method was direct oblimin. Confirmatory factor analysis confirmed the new structure. Finally, Cronbach's alpha internal consistency coefficient was calculated. The accepted significance level was 0.05 for a 95% confidence interval.

## Results

### Calculation of the content validity index

The Turkish version of the scale was presented to 13 nursing management and internal medicine nursing specialists. As a result of the analysis using the Davis technique, the items' content validity ratios (CVR) ranged from 0.85–1. Therefore, the content validity index of the scale was 0.96 after the content validity rate of each item was summed and divided by the total number of items obtained. The Turkish version was then translated into English by two academicians, one a medical doctor and the other a nurse with a Ph.D.

### Performing item-total score correlation analyzes to reveal the compatibility between the items

Correlation values obtained from item-total correlation analyses with 38 items regarding the Turkish version of the QNC are shown in Table 2. As a result of the investigation, the item-total score correlation coefficients of the items differed between *r* = 0.72 and 0.92.

**Table 2:** Content validity ratios, item total point correlation values and factor loadings of the items

Item no	CVR	r	FL	Item no	CVR	r	FL
**1**	**1**	**.72**	**.72**	20	1	.87	.88
**2**	**1**	**.73**	**.72**	21	1	.87	.88
**3**	**1**	**.72**	**.72**	22	1	.85	.86
**4**	**1**	**.75**	**.76**	23	.92	.81	.82
**5**	**1**	**.73**	**.73**	24	.92	.85	.87
**6**	**.85**	**.74**	**.74**	25	1	.89	.90
7	1	.86	.86	26	1	.83	.85
8	1	.86	.87	27	1	.87	.88
9	.92	.82	.83	28	.85	.89	.86
10	.92	.86	.87	29	.85	.90	.84
11	.92	.87	.88	30	.92	.90	.82
12	1	.87	.88	31	.92	.92	.88
13	.92	.86	.87	32	1	.91	.84
14	1	.86	.87	*33*	*1*	*.81*	*.57*
15	.92	.87	.88	*34*	*1*	*.87*	*.58*
16	1	.86	.87	*35*	*1*	*.88*	*.56*
17	.85	.87	.89	*36*	*1*	*.85*	*.42*
18	.92	.86	.87	*37*	*1*	*.82*	*.52*
19	.92	.87	.88	*38*	*1*	*.84*	*.46*

*CVR* Content validity ratio, *r* Item total point correlation value, *FL* Factor loading

### Factor analysis to reveal construct validity

The KMO value was high at 0.975, and Bartlett’s test was significant at the < 0.001 level. Then, confirmatory factor analysis was performed first. However, the × 2/df value was 9.82 for the six factored structures. Then an explanatory factor analysis was made to understand the new structure in the Turkish language. The results showed that the factor loads of the items ranged from 0.42 to 0.93. Three subscales explaining 80.806% of the total variance were revealed. First, items 4, 5, 6, 7, 8, 9, and 10 were cross loaded to factors 1 and 2. Next, items 24, 26, and 27 crosses loaded in Factor I and 3.

However, the differences in the factor load values of the same items in different factors were over 0.300. Therefore, those items remained in the factors that got higher factor loads.

**Fig. 1:**
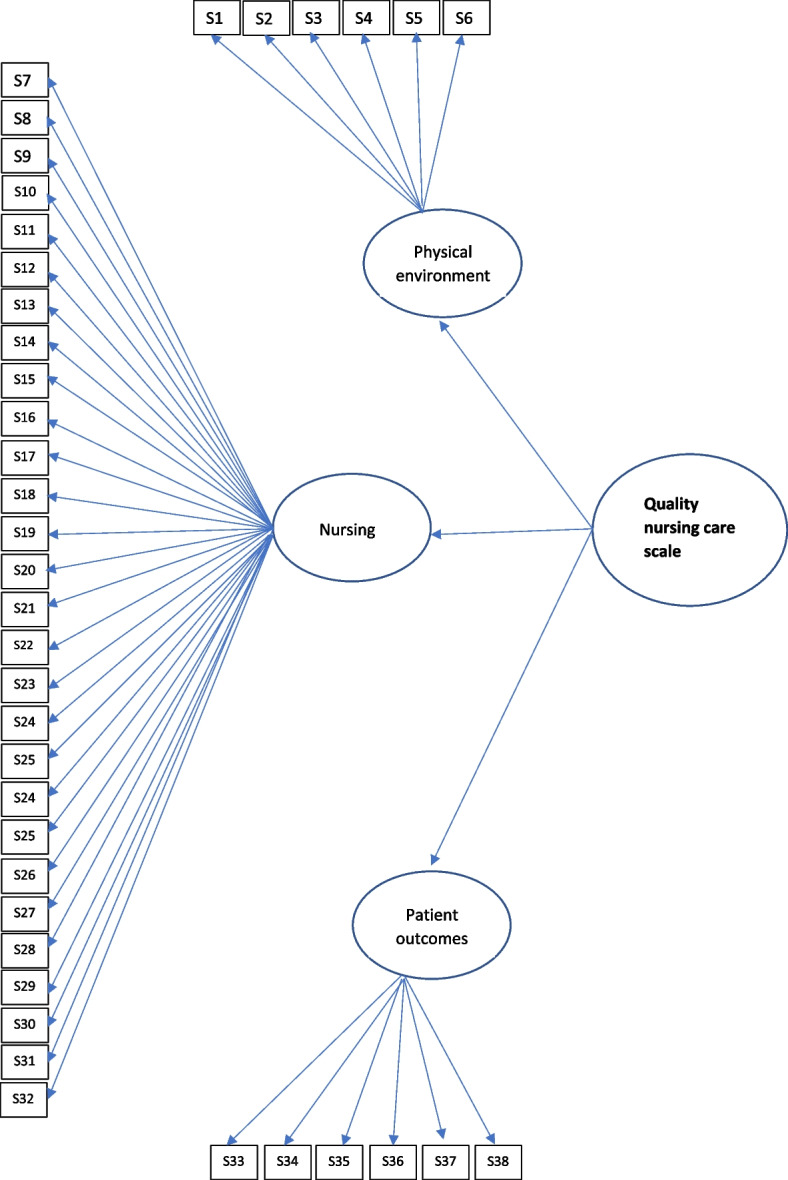
Path diagram of confirmatory factor analysis

Then confirmatory factors analysis was repeated for the new structure, and the × 2/df value was calculated as 3.85 (Table 3). The Comparative Fit Index, Standardized Root Mean Square Residual and the Root Mean Square Error of Approximation indices were 0.90, 0.041, and 0.011, respectively (Fig. 1).

**Table 3:** Fit indices for the confirmatory factor analysis

**Fit indices and x**^**2**^**/df values**	**CFI**	**SRMR**	**RMSEA**
	0.90	0.0407	0.011
**Acceptable Fit Values**	> 0.90	< .080	< .080
**Good Fit Values**	> 0.95	< 0.080	< 0.050
**x**^**2**^	2408		
**df**	626		
**x**^**2**^**/df**	3.85		
**Acceptable value for x**^**2**^**/df**	< 5		
**Good value x**^**2**^**/df**	< 2		

*CFI* The comparative fit index, *SRMR* Standardized root mean square residual, *RMSEA* The root mean square error of approximation, *df* degree of freedom

### Determination of scale internal consistency coefficient for reliability analysis

Cronbach’s alpha coefficient of the Turkish version of the Scale was 0.99. The same coefficient for the first subdimension was 0.95, and there were 0.99 for the second and third subdimensions (Table 4).

**Table 4:** Item total point correlation values of the items

Original version	Cronbach’s alpha	Adapted version	Cronbach’s alpha
Physical environment	.90	Physical environment	.958
Staff characteristics	.92	Nursing	.99
Precondition	.88		
Task orientated activities	.88		
Human orientated activities	.89		
Patient outcomes	.85	Patient outcomes	.99
Quality Nursing Care Scale	.96	Quality Nursing Care Scale	.99

## Discussion

A limited number of measurement tools evaluating nursing care quality requires the development or adaptation of valid and reliable tools assessing the quality of nursing care. Unfortunately, only two measurement tools have been conducted on this subject. One of them is the Caring Behaviors Inventory-24 [25]. The scale was designed to evaluate the nursing care process/quality. Another scale was developed by Leinonen et al. to assess the perceptions of patients’ quality of perioperative care [26]. Lennon et al. [27] made minor changes to the items in the scale so that they could be applied to nurses and patients. This study aims to perform psychometrics of Liu et al.’s The Quality of Nursing Care Scale, which consists of 38-item and evaluates the quality of nursing care in the aspect of nurses [10].

Evaluating the quality of care, nurses provide data that will help prevent errors, minimize possible harm to patients, and identify risky situations. At this stage, the validity and reliability of the Turkish version of the QNC are discussed under the headings of language validity, content validity, item-total score correlation analysis, construct validity, and internal consistency reliability.

### Language validity

Since the translated items might not mean the same in the adapted language [23, 28], experts were asked to evaluate the restated items in terms of meaning. Minor revisions were made considering the experts' recommendations to clarify their meanings in Turkish. For example, wording arrangements were made for Item 5 (I provide a quiet ward environment for patients staying in the hospital), Item 14 (I work well with my team [other nurses and healthcare providers]), Item 16 (I master the clinical, technical operations to meet the needs of nursing care), Item 20 (I can manage drugs well), and Item 21 (I intend to help patients whenever the help is needed). Then, two academicians who knew both languages backtranslated the items into English.

### Content validity

Davis technique, a frequently used method developed by a nurse researcher, was used for the content validity analysis [29]. The original and Turkish items were presented to the experts and asked to compare based on their meanings and grammatical structure. It was observed that the experts mainly assessed the Turkish items in the scale as “quite appropriate.” The lowest CVR value was 0.80, an acceptable value in the literature [29, 30].

### Evaluation of correlations between items

This study evaluated the compatibility of 38 items using item-total score correlations. It was seen that the correlation, or coherence, of each item of the scale with the whole scale.

### Examination of construct validity

Although it is recommended that the scale's construct validity be evaluated using confirmatory factor analysis for adaptation studies [31], this study used explanatory factor analysis because the original model did not fit in Turkish based on the confirmatory factor analysis results. Since the literature indicated that the scale's construct would not have the same structure in the local context [23, 32], the authors decided to perform a new explanatory factor analysis to explore the system in Turkish. Unlike in the original study, the items were distributed into three subscales instead of six (Table 5). Only the Physical environment and Patient outcomes subscales remained the same. Staff characteristic, Precondition, Task-orientated activities, and Human-orientated activities subscales merged into one subdomain in the Turkish context. When the statements were evaluated carefully, the meanings of the statements were precise in Turkish. However, it was assessed that nurses perceived the statements under one subdomain because they perceived all items under Staff characteristic, Precondition, Task-orientated activities, and Human-orientated activities subscales related. The authors evaluated that those items differed from others because they were mainly associated with primary nursing roles and tasks. For example, the items in the physical environment subdomain might also be related to the other staff and the hospital environment. Patient outcomes were related to the customers' perspective. However, the items that emerged with one subdomain were directly associated with nurses and nursing. Therefore, the authors named that domain "Nursing."

**Table 5:** Distrubiton of the items in the original work and Turkish Version

Distrubution of the item in the original work	Distrubution of the item in the Trukish form
***Physical environment (6 items)***	***Physical environment (6 items)***
I provide the hygienic room to the patients	I provide the hygienic room to the patients
I provide a comfortable environment for patient to rest in	I provide a comfortable environment for patient to rest in
I keep patient room has the good ventilation	I keep patient room has the good ventilation
I provide safe environment to patients for their treatment	I provide safe environment to patients for their treatment
I provide the quiet ward environment for patients staying in the hospital	I provide the quiet ward environment for patients staying in the hospital
I can immediately dispose patients’ reflection environment problems	I can immediately dispose patients’ reflection environment problems
***Staff characteristic (8 items)***	***Nursing (26 items)***
I am very cautious in performing my nursing duties	I am very cautious in performing my nursing duties
I carefully follow hospital rules and regulations	I carefully follow hospital rules and regulations
I closely observe the patient condition, focusing on the dynamic change of the disease	I closely observe the patient condition, focusing on the dynamic change of the disease
I am polite and pleasant to treat patients	I am polite and pleasant to treat patients
I smile to patients when providing nursing service	I smile to patients when providing nursing service
I patiently listen to my patients, when they want to talk about their problems	I patiently listen to my patients, when they want to talk about their problems
I patiently and repeatedly explain patients doubt	I patiently and repeatedly explain patients doubt
I work well with my team (other nurses and healthcare providers)	I work well with my team (other nurses and healthcare providers)
***Precondition (7 items)***	
I can up-to-data my theoretical knowledge to meet the needs of nursing care	I can up-to-data my theoretical knowledge to meet the needs of nursing care
I master the clinical technical operations to meet the needs of nursing care	I master the clinical technical operations to meet the needs of nursing care
I master operating process of basic nursing care and special nursing care	I master operating process of basic nursing care and special nursing care
My professional experience is helpful for my nursing job	My professional experience is helpful for my nursing job
I participate in the ward quality management	I participate in the ward quality management
I can manage drugs well	I can manage drugs well
I intend to help patients whenever the help is needed	I intend to help patients whenever the help is needed
***4. Task-oriented activities (6 items)***	
I provide sufficient information related to nursing care to patients’ relatives	I provide sufficient information related to nursing care to patients’ relatives
I can explain clearly to the patients about their questions related to medical expense related to nursing care	I can explain clearly to the patients about their questions related to medical expense related to nursing care
I provide guidance to do self-care for my patients	I provide guidance to do self-care for my patients
I perform the good basic nursing care to patients	I perform the good basic nursing care to patients
I provide individualized care for patients	I provide individualized care for patients
I provide effective health education for patients	I provide effective health education for patients
***5. Human-oriented activities (5 items)***	
I can analyze the patient psychological feelings to provide care	I can analyze the patient psychological feelings to provide care
I provide humanity services to patients based on their characteristics	I provide humanity services to patients based on their characteristics
I can help patients build confidence to overcome the disease	I can help patients build confidence to overcome the disease
I help my patients to relieve their fear about treatment and procedure	I help my patients to relieve their fear about treatment and procedure
I help my patients to relieve their worry about illness	I help my patients to relieve their worry about illness
***6. Patient outcomes (6 items)***	***6. Patient outcomes (6 items)***
I have never get complains from the patients and their relatives	I have never get complains from the patients and their relatives
I ensure to provide service would meet patient's satisfaction criteria	I ensure to provide service would meet patient's satisfaction criteria
I ensure to provide safety service to patient	I ensure to provide safety service to patient
I can avoid patient physical damage (such as fall, burn, and pressure sore)	I can avoid patient physical damage (such as fall, burn, and pressure sore)
I can avoid patient chemical damage (such as drug misuse, drug incompatibility, and wrong medication)	I can avoid patient chemical damage (such as drug misuse, drug incompatibility, and wrong medication)
I can avoid patient biological damage (such as bacterium, virus, and fungus infection)	I can avoid patient biological damage (such as bacterium, virus, and fungus infection)

### Internal consistency analysis

The Cronbach's alpha internal consistency test, the most commonly used test to assess the internal consistency of Likert scales, evaluated the reliability of the subscales and scale in Turkish [33]. Although the acceptable Cronbach's alpha coefficient level has varied in previous reports, the commonly recommended threshold value is 0.70 [34–36]. The Cronbach's alpha coefficients of the scale and subscales in Turkish form were relatively high and indicated high reliability. The coefficients were also higher than the original work's values.

### Limitations

Although we planned to reach out more participants and to perform confirmatory and exploratory factor analysis on different sample groups in study setting, we could not reach out enough number of nurses because of the huge working conditions during COVID-19 Pandemic.

## Conclusions

The study concluded that Turkish version of the QNC was a valid and reliable tool among clinical nurses. The psychometrics characteristics of the form revealed that the tool had required qualifications and could be used to evaluate nurses’ perceptions of the quality of nursing care in studies conducted in Turkey.

Nurse managers can identify institutional-level problems using the results of nurses’ evaluation of the quality of care provided and develop quality improvement programs in this direction. Owing to these practical strategies, cost savings and optimal maintenance can be achieved. In addition, researchers studying topics related to the subject can benefit from the scale in evaluating nursing care from the nurses’ perspective. Since stability was not evaluated in this study, further studies may measure it.

## Data Availability

The data that support the findings of this study are available from the corresponding author upon reasonable request.

## Abbreviations

QNC: Quality nursing care scale
CVR: Content validity ratio
KMO: Kaiser–Meyer–Olkin test
